# Solution for Survey Discrepancies in Washington State Smoking Prevalence

**Published:** 2005-12-15

**Authors:** Michael J Boysun, Julie E Maher, Michael J Stark, Barbara A Pizacani, Kristen Rohde, Julia Dilley, Katrina Wynkoop Simmons

**Affiliations:** Tobacco Prevention and Control Program, Washington State Department of Health, Olympia, Wash; Program Design and Evaluation Services, Multnomah County Health Department, Oregon Department of Human Services, Portland, Ore; Program Design and Evaluation Services, Multnomah County Health Department, Oregon Department of Human Services, Portland, Ore; Program Design and Evaluation Services, Multnomah County Health Department, Oregon Department of Human Services, Portland, Ore; Program Design and Evaluation Services, Multnomah County Health Department, Oregon Department of Human Services, Portland, Ore; Tobacco Prevention and Control Program, Steps to a HealthierUS Washington State, Olympia, Wash; Behavioral Risk Factor Surveillance System Coordinator, Washington State Department of Health, Olympia, Wash

## To the Editor:

Consistent with the findings of Ramsey et al (1), we found that the Washington State smoking prevalence data from the Adult Tobacco Survey (ATS) were lower than the prevalence data from the Behavioral Risk Factor Surveillance System (BRFSS). In this letter, we discuss how Washington resolved this problem of disparate prevalence estimates and still obtained population-based survey data on many tobacco-related measures.

Although the BRFSS is conducted in Washington to collect data on health behaviors, including tobacco-related health behaviors (2), the Washington Tobacco Prevention and Control Program also conducted the ATS from 2000 through 2002 to obtain extensive information on tobacco-related knowledge, attitudes, and behaviors. Like the New Hampshire ATS and BRFSS, the Washington ATS and BRFSS were both random-digit–dialed statewide telephone surveys of noninstitutionalized adults that used the same questions to measure tobacco prevalence, and both surveys had similar response rates (Table). However, the ATS contained strong, tobacco-specific introductory language, and the BRFSS contained general, health survey introductory language. For each year from 2000 through 2002, the ATS found a lower smoking prevalence in Washington State than did the BRFSS, and this difference became statistically significant in 2001 and 2002 (Table). The lack of a significant difference between the ATS and BRFSS findings in 2000 was likely a result of survey estimate variability related to the smaller BRFSS sample sizes. Therefore, our results support the conclusion of a California study by Cowling et al that the tobacco-specific survey introduction is associated with underreported tobacco use by some smokers (3). Cowling et al state: "The specificity of the introduction may cue respondents to adjust their responses (i.e., deny tobacco use) in order to shorten the length of the interview experience" and "provide a socially desirable response." Cowling et al do not provide additional information on whether the order of the smoking questions might have also contributed to this difference in prevalence, a possibility suggested by Ramsey et al (1).

In 2003, Washington began incorporating ATS questions into the BRFSS, partly to prevent this apparent underreporting in the ATS. Using a modular approach similar to that used for Oregon's BRFSS, we created an instrument that meets the needs of general public health surveillance tools (e.g., the BRFSS) as well as tobacco-related surveillance tools (e.g., the ATS). In our instrument, the general health survey introduction from the BRFSS is used for all survey respondents. Respondents answer the core demographics and health questionnaire of the BRFSS and then either a module of state-specific questions or a module of tobacco-specific questions, many of which are from the ATS. The average survey length of each module is about the same as the length of each module in the 2002 BRFSS survey.

Smoking prevalence based on the expanded BRFSS data for 2003 (N = 18,644) was 19.8% (95% confidence interval [CI], 19.2%–20.6%), which was more similar to previous BRFSS prevalence estimates than previous ATS estimates (Table). This finding was reassuring and suggested that the presence of numerous tobacco-related questions on the BRFSS did not create a bias similar to that generated by a tobacco-specific introduction.

In addition to providing potentially less-biased surveillance data, the modular approach to the BRFSS provides additional benefits. First, more states conduct the BRFSS than the ATS, so more comparisons of results can be made, and unlike the ATS the BRFSS is conducted throughout the year. Second, our modular approach has tripled the size of the core BRFSS questionnaire, enabling the tobacco-control program and other programs to perform more subgroup analyses. Third, this approach facilitates examination of associations between tobacco-related measures and other health indicators. Fourth, more room on the survey is available on the nontobacco module for other programs to add questions. Finally, the modular approach streamlines the surveillance by saving staff time and minimizing the time required of participants. Using procedures and protocols developed for the BRFSS incorporates oversight expertise for both surveys into one operation.

## Figures and Tables

**Table:** Washington State Smoking Prevalence Rates Based on the Adult Tobacco Survey (ATS) and Behavioral Risk Factor Surveillance System (BRFSS), 2000–2002

**Year**	**ATS^a^**		**BRFSS^a^**		***χ* ^2^_1_ (*P*)^c^**

	**Current Smokers, No. (%)**	**95% CI^b^**	**Current Smokers, No. (%)**	**95% CI^b^**	
2000	1783 (18.9)	17.6-20.2	746 (20.7)	19.3-22.3	3.5 (.06)
2001	2093 (17.0)	16.0-18.0	939 (22.5)	21.1-23.9	39.7 (<.001)
2002	1432 (16.1)	15.2-17.0	969 (21.5)	20.0-23.0	39.4 (<.001)

a The Council of American Survey Research Organizations (CASRO) response rates were generally lower for the ATS (35%–40%) than for the BRFSS (40%–50%)

b CI indicates confidence interval.

c The design-based *F* statistic was used to calculate the chi-square value.
